# Evidence across CMR sites and systems of background velocity offset errors requiring correction before accurate measurement of regurgitant and shunt flow

**DOI:** 10.1186/1532-429X-11-S1-O96

**Published:** 2009-01-28

**Authors:** Peter D Gatehouse, Marijn P Rolf, Martin J Graves, John Totman, Jochen von Spiczaki, Maria-Filomena Santarelli, Yingmin Liu, Rebecca A Quest, Matthias Dieringer, Massimo Lombardi, Jürg Schwitter, Jeanette Schulz-Menger, David N Firmin, Mark BM Hofman, Philip J Kilner

**Affiliations:** 1 Royal Brompton Hospital, London, UK; 2 VU Medisch Centrum, Amsterdam, Netherlands; 3 Cambridge University Hospitals NHS Foundation Trust, Cambridge, UK; 4 King's College, London, UK; 5 Institute for Biomedical Engineering, University and ETH, Zurich, Switzerland; 6 CNR Institute of Clinical Pharmacology, Pisa, Italy; 7 University of Auckland, Auckland, New Zealand; 8 Imperial College Healthcare NHS Trust, London, UK; 9 Franz-Volhard-Klinik, Charité Universitätsmedizin, Berlin, Germany; 10 University Hospital, Zurich, Switzerland

**Keywords:** Flow Image, Shunt Flow, Pulmonary Regurgitation, Scanner Type, Protocol File Transfer

## Purpose

To assess velocity offsets in the background of phase-contrast acquisitions across CMR sites and systems.

## Introduction

Phase-contrast CMR potentially provides accurate measurements of aortic or pulmonary regurgitation, cardiac output and shunt flow. Among several known errors (assuming concomitant gradient correction [1]) we examined one: a 2 cm/s velocity offset (i.e. around 1% of a typical VENC) can cause >20% error in cardiac output [2, 3] with larger consequences for regurgitation and shunt flow, particularly in dilated vessels.

As a collaborative group, we measured velocity offsets across sites and scanner types, an initiative backed by the European Society of Cardiology CMR Working Group.

## Methods

**Figure 1:**
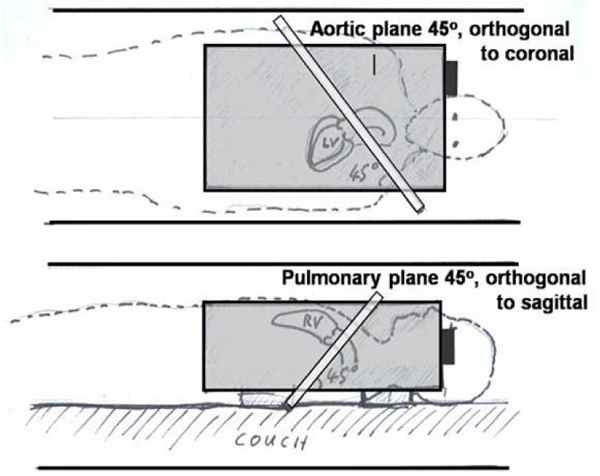
**The planes used for veolicity offset acquisitions at all sites**. The aortic (AO) plane at 45 degress between transaxial and sagittal used antero-posterior phase-encode. The main pulmonary artery (MPA) plane at 45 degrees between transaxial and coronal was repeated with head-foot and left-right phase-encoding. The grey bloc represents the gelatin phantom.

To eliminate slow flow, 10–15 litre uniform gelatine phantoms were used, containing 5 millimoles/litre Gd-DTPA for SNR. We used similar phase-contrast sequence parameters at each site, measuring 45° oblique 'aortic' (Ao) and 'main pulmonary artery' (MPA) planes (Figure 1). Constant sequence parameters were: retro-gated cine at 1000 ms RR-interval, through-plane Venc = 150 cm/s, SLT = 6 mm, TE = 2.8–3.0 ms, FOV = 320 mm square, uninterpolated pixels 1.25 mm(FE) by 2.5 mm(PE), bandwidth 355 Hz/pixel, 6 rawdata lines per cardiac cycle, no cine data-sharing or parallel imaging. Unless stated, velocity encoding was asymmetric (i.e. phase-subtraction of compensated and velocity-encoded). The gradient-echo was asymmetric (early for short TE); Philips applied partial-echo sampling which may explain its shorter TR. Slower machines were excluded because fast gradient performance was necessary. Three 1.5 T scanner types were compared, and the sequences were reproduced exactly among the 4 sites of each scanner type by protocol file transfer (except 1 site below):

GE (4 sites) Signa Excite 14M5. Symmetric velocity-encoding (compulsory), flow analysis on, flow optimization off, TR5.9–6.0 ms, TE2.9–3.0 ms (image orientation dependent) (1 site on 12M5 TR5.7–5.8 ms TE2.9 ms)

Philips (4 sites) Achieva R2.53. TR5.5 ms, TE2.8 ms, asymmetric RF pulse (late centre), phase correction off.

Siemens (4 sites) Avanto VB15 TR6.6 ms, TE2.8 ms.

Velocity images were reconstructed without offset correction (which performed unrealistically well in this large uniform phantom). Within 50 mm of isocentre, the largest offset in cm/s averaged over 300 mm^2^ circular ROI was recorded for each plane. All images were measured independently by two sites; total error was estimated between these, also from noise and cine frame variations.

## Results

Please see data in Table 1.

**Table 1:** Largest ROI mean offset (cm/s) within 50 mm of Isocentre, for aortic slice, MPA slice (HF phase-encoding) and MPA slice (LR phase-encoding). The four rows per slice are from the four sites using each scanner type. The column order is not specified, i.e. scanner types are not identified. (< ± 0.3 cm/s stdev total error as described above)

cm/s	Site	Scanner type 1	Scanner type 2	Scanner type 3
Aorta	1	2.5	1.4	1.7
	2	0.7	2.5	0.9
	3	0.9	2.9	1.4
	4	1.9	1.6	0.7
MPA (HF phase-enc)	1	0.8	3.9	1.0
	2	1.1	3.4	1.3
	3	0.8	4.9	1.2
	4	1.0	3.9	0.8
MPA (LR phase-enc)	1	1.2	3.3	1.5
	2	1.0	5.3	1.6
	3	0.8	5.0	1.8
	4	0.4	3.6	0.6
Maximum per site	1	2.5	3.9	1.7
	2	1.1	5.3	1.6
	3	0.9	5.0	1.8
	4	1.9	3.9	0.8

## Discussion and conclusion

The results are believed reliable for 3 reasons: 1) cine images were stable without ghosting, 2) similar results occurred in nearby parallel slices, 3) image analysis was repeated independently. Comparison of hardware is prevented by remaining differences between sequences; we emphasise that small sequence changes may alter these results. An offset of 1% of Venc (π/100) is impressive engineering, representing a residual gradient of ≈0.02% (for approx. TE/2, 50 mm from isocentre) of typical velocity-encoding gradients. This extreme sensitivity to adjustments such as pre-emphasis could explain variations between nominally identical sites.

Various automatic offset corrections are routinely installed. However, this study intentionally omitted them; their usefulness may depend on applications. Offset correction uses stationary tissue pixels which can be identified automatically based on their smaller temporal variation [2]), or identified by users during postprocessing. This approach is sometimes limited by insufficient stationary tissue, its low SNR in flow images, and possible spatial non-linearity. A more time-consuming approach repeats identical flow acquisitions on a static phantom, subtracting the corresponding apparent phantom velocities from the clinical acquisition [2].

We conclude that most systems require velocity offset correction of flow images for the most sensitive clinical applications. There is a general need for optimization of acquisition protocols or (if possible) system engineering and correction methods to minimise velocity offsets.
